# Molecular Signatures Underlying Synaptic Vesicle Cargo Retrieval

**DOI:** 10.3389/fncel.2017.00422

**Published:** 2018-01-05

**Authors:** Yasunori Mori, Shigeo Takamori

**Affiliations:** Laboratory of Neural Membrane Biology, Graduate School of Brain Science, Doshisha University, Kyoto, Japan

**Keywords:** synaptic vesicles, endocytosis, clathrin, endocytic motif, pHluorin

## Abstract

Efficient retrieval of the synaptic vesicle (SV) membrane from the presynaptic plasma membrane, a process called endocytosis, is crucial for the fidelity of neurotransmission, particularly during sustained neural activity. Although multiple modes of endocytosis have been identified, it is clear that the efficient retrieval of the major SV cargos into newly formed SVs during any of these modes is fundamental for synaptic transmission. It is currently believed that SVs are eventually reformed via a clathrin-dependent pathway. Various adaptor proteins recognize SV cargos and link them to clathrin, ensuring the efficient retrieval of the cargos into newly formed SVs. Here, we summarize our current knowledge of the molecular signatures within individual SV cargos that underlie efficient retrieval into SV membranes, as well as discuss possible contributions of the mechanisms under physiological conditions.

## Introduction

Synaptic vesicles (SVs) are the storage organelle of neurotransmitters. The arrival of an action potential elicits the release of neurotransmitter from SVs via exocytosis. After exocytosis, the constituents of SVs, including lipids and integral membrane proteins, are retrieved by a process called endocytosis and recycled for the next round of exocytosis (Südhof, 2013). Thus, even without a supply of proteins and lipids from the cell body, SVs can be locally recycled and reused at presynaptic terminals within a certain period. Several distinct modes of SV membrane retrieval have been proposed, largely based on electron microscopy analysis (reviewed by Soykan et al., 2016). Upon relatively mild stimulation, such as 10 Hz, SV membranes are recycled by clathrin-mediated endocytosis (CME), a process that involves the formation clathrin-coated pits from the plasma membrane at active zones (AZs) or from cisternal structures at the periphery of the release sites. With stronger stimulation (e.g., 40 Hz, 5–10 s), formation of much larger membranous structures known as endosome-like vacuoles (ELVs) or activity-dependent bulk endocytosis (ADBE) occurs; the structures formed are not decorated with clathrin coats (Clayton et al., 2008; Kononenko et al., 2014). Furthermore, clathrin-independent ultrafast endocytosis (UFE) has also been proposed to occur predominantly at physiological temperatures, in which endosomes emerge immediately from the plasma membrane after a brief stimulus (<100 ms) (Watanabe et al., 2013). In the case of the latter two processes, SVs are subsequently regenerated from the endosomes in a clathrin-dependent manner (Watanabe et al., 2014). Although another type of rapid SV retrieval called “kiss-and-run” exocytosis and endocytosis, involving transient fusion pore formation and subsequent closure, has been observed in neuroendocrine cells, controversy exists regarding whether the same mechanism exists in neurons (Aravanis et al., 2003; Granseth et al., 2006).

Because clathrin itself cannot directly interact with lipids or various cargo proteins, adaptor proteins play crucial roles in recruiting the clathrin coat to the cluster sites of the SV cargo to be endocytosed (Traub and Bonifacino, 2013; Paczkowski et al., 2015). These adaptors can directly bind to SV cargo proteins, while simultaneously recruiting the clathrin coat proteins for the initiation of SV formation, either from the plasma membrane or endosomes pinched off from the plasma membrane. Among adaptor proteins, AP-2 (a heterotetramer complex composed of α, β2, μ2, and σ2 subunits) might play a central role in the recognition of SV cargos at the plasma membrane, enabling the selective retrieval of SV cargo proteins and preventing unexpected contamination of plasma membrane residents. Other adaptor protein complexes, such as AP-1 and AP-3, may play similar, but distinct, roles in the recognition of SV cargos at different intracelluar sites; e.g., endosomes (Kokotos and Cousin, 2015). Further, other accessory proteins specific for certain SV cargos, such as AP-180/clathrin assembly lymphoid myeloid leukemia (CALM) family, endophilin, and stonin, likely contribute to cargo selection and efficient retrieval. In addition to specialized adaptors that link SV cargos to clathrin, specific or non-selective cargo–cargo interactions within SVs may contribute to selective cargo retrieval into newborn SVs. The latter mechanism can be particularly important and economic in maintenance of the stoichiometry of the major SV components.

In this review, we will first discuss how the faithful retrieval and maintenance of SV cargo proteins would impact synaptic transmission. We will then discuss various molecular signatures within SV cargo proteins that enable efficient sorting into newborn SVs, which have been identified by pHluorin-based probes. Moreover, we will consider several endocytic pathways through which large intermediate compartments are involved to generate competent SVs. Descriptions of various modes of endocytosis, i.e., presynaptic membrane retrieval and SV reformation, can be found in other recently published reviews (e.g., Kononenko and Haucke, 2015; Soykan et al., 2016).

## Major SV cargos and the physiological importance of their faithful retrieval

SVs are equipped with dozens or more proteins that are responsible for basic synaptic functions, such as neurotransmitter uptake and exocytosis, as well as for activity-dependent changes underlying synaptic plasticity. Quantitative molecular analysis of purified SVs from rodent brains demonstrated that SVs are “protein-rich” supramolecular complexes, with approximately a quarter of membrane volume occupied by the transmembrane helices conferred by abundant membrane proteins (Takamori et al., 2006). These proteins include the following: (1) synaptobrevin 2 (Syb2; also referred to as VAMP2), which is essential for the fusion of the SV membrane with the plasma membrane; (2) synaptotagmin 1 (Syt1), which functions as a Ca^2+^ sensor for exocytosis; (3) vesicular transporters that allow the SV lumen to be filled with neurotransmitters, such as glutamate and GABA; and (4) other membrane proteins (SV2 and synaptophysin) with functions that remain elusive. Although in mammals, these SV proteins consist of multiple isoforms, which are usually expressed in a subpopulation of neurons, the average copy numbers of the abundant isoforms in an SV can vary substantially (Table 1), with Syb2 being the most abundant protein (30–70 copies per SV) and the vacuolar-type H^+^-ATPase (V-ATPase) being the least abundant protein complex (1–2 complexes per SV) (Takamori et al., 2006; Mutch et al., 2011; Wilhelm et al., 2014). Moreover, there appears to be variation in the protein number per vesicle. Some SV proteins, such as SV2, V-ATPase, Syt1 and the vesicular glutamate transporter 1 (VGLUT1), show very little variability in the protein number between vesicles, whereas Syp, Syb2, and synaptogyrin show significant intervesicle variability (Mutch et al., 2011). In addition to the forementioned major SV constituents, SVs contain various integral membrane proteins, such as endosomal soluble NSF attachment protein receptors (SNAREs), which seem to be present on only a subset of SVs. The acquisition of these “minor” proteins during recycling may confer distinct release properties on individual SVs (Hoopmann et al., 2010; Hua Z. et al., 2011; Raingo et al., 2012; Ramirez et al., 2012).

**Table 1:** Endocytic motif in the major SV cargos.

**Name**	**Abbreviation**	**Function**	**Copy No./SV**	**Endocytic motif**
Synaptobrevin2/VAMP2	Syb2 or VAMP2	Catalysis of membrane fusion	32^(1)^, 70^(2)^	PRD,SNARE motif, di-leucine motif
Synaptotagmin 1	Syt1	Ca^2+^ sensor for synchronous release	7^(1)^, 15^(2)^	Basic motif in C2 domain, di-leucine motif
Synaptophysin	Syp	Unknown	13^(1)^, 30^(2)^	Tyrosine repeats
Synaptic vesicle protein 2A	SV2A	Unknown	2^(1)^, 5^(2)^	Tryrosine-based motif
Vesicular glutamate transporter 1	VGLUT1	Glutamate uptake	4^(1)^, 10^(2)^	PRD, di-leucine motif
Vesicular GABA transporter	VGAT/VIAAT	GABA uptake	N.D.	di-leucine motif

*Functions in neurotransmission, average copy numbers per SV and endocytic motifs within the amino acid sequences are listed. Copy numbers were taken from ^(1)^Mutch et al or ^(2)^Takamori et al. respectively. N.D. stands for “not determined”. In general, copy numbers of SV proteins estimated in ^(1)^are smaller than those reported in ^(2)^. Although both measurements relied on the usage of specific antibodies for each SV protein,anybody labeling in ^(1)^was performed in situ where accessibility of antibodies to the epitopes could be limited because of both crowding of the epitopes and possible protein-protein interactions that can mask the epitopes in native vesicles, leading to underestimation of the copy numbers*.

With the exception of V-ATPase, essential proteins for exocytosis and transmitter uptake appear to have sufficient “safety margins,” which ensure that sufficient copies of these components are always present during SV recycling, even if some proteins fail to be incorporated. For instance, only a few copies of Syb2 have been shown to be required for rapid exocytosis (Mohrmann et al., 2010; van den Bogaart et al., 2010; Sinha et al., 2011). Thus, sustained neurotransmission does not require the precise retrieval of Syb2 during recycling. Consistent with this notion, previous research on Syb2^+/−^ mice failed to identify noticeable electrophysiological phenotypes with regard to synaptic transmission (Schoch et al., 2001). However, recent data on Syb2^+/−^ hippocampal slice preparations revealed reduced basal transmission and altered release probability (Koo et al., 2015), underscoring the importance of a precise mechanism to ensure the presence of sufficient copies of Syb2 on SVs during recycling. Similarly, mice heterozygous for the vesicular glutamate transporters (VGLUT1 and VGLUT2) exhibited a wide array of behavioral deficits despite little electrophysiological phenotype (Takamori, 2006; Tordera et al., 2007; Schallier et al., 2009; Elizalde et al., 2010). Therefore, even for the major SV constituents, a mechanism must exist to ensure the faithful retrieval of SV cargo proteins to generate SVs functionally competent to maintain the fidelity of neurotransmitter uptake and SV exocytosis during high activity.

## Visualizing SV cargo retrieval

A green fluorescent protein derivative with enhanced pH sensitivity (pHluorin) has long been used to monitor SV dynamics in cultured neurons and has also enabled the characterization of individual SV cargo dynamics upon various forms of stimulation (Kavalali and Jorgensen, 2014). When the luminal domain of an SV cargo protein is tagged with a pHluorin moiety and the fusion protein is exogenously expressed in cultured neurons, the fluorescence of the pHluorin probes is quenched in resting conditions owing to the acidic environment of the SV lumen. When a stimulus is applied to neurons and exocytosis occurs, pHluorin molecules are exposed to the extracellular (neutral) solution and, therefore, the fluorescence of the probe is consequently de-quenched. Thereafter, endocytosis and the subsequent acidification after (and during) the stimulus re-quench the fluorescence. According to the schematic view of the endocytosis process, initial studies using a prototype of a pHluorin probe called synaptopHluorin (pHluorin is C-terminally tagged to Syb2) were interpreted based on a premise that the optical probes share a common destiny with all other endogenous SV cargo proteins and lipid constituents (Miesenböck et al., 1998; Sankaranarayanan and Ryan, 2000; Fernandez-Alfonso and Ryan, 2004). However, subsequent studies, in which the luminal regions of other SV proteins, such as Syt1, VGLUT1, Syp, and SV2, were tagged with similar pHluorin probes, revealed that this premise did not always hold true (Pan et al., 2015), indicating that the retrieval of individual SV cargo might be differentially regulated by multiple factors.

One of the intrinsic problems associated with the use of pHluorin-probes to track SV cargo is that fluorescence signals do not directly report the dynamics of SV cargos, but depend on the re-acidification kinetics of the endocytosed vesicle lumen. Although the pKa of the most frequently-used pHluorin (known as superecliptic pHluorin: SEP) is better suited to minimize any contribution of the re-acidification process during CME (Atluri and Ryan, 2006; Granseth et al., 2006), re-acidification kinetics can be rate-limiting when large vacuoles are generated by ADBE or UFE, owing to their large volume (Nicholson-Fish et al., 2015; Okamoto et al., 2016). Therefore, since a wide range of stimulation protocols have been used to monitor SV cargo recycling, ranging from 5 to 80 Hz, when multiple modes of endocytosis could be elicited, it should be noted that pHluorin signals may include multiple reporter statuses in various endocytosed vesicles upon repetitive stimulation.

## Dynamics of SV cargo at the plasma membrane

A method to maintain the stoichiometry of all SV cargo proteins during exocytosis and endocytosis is to avoid the dispersion of vesicle proteins in a single vesicle, presumably via relatively tight protein–protein interactions among the cargo proteins. High-resolution imaging of Syt1 immunostaining by stimulated emission depletion microscopy revealed that Syt1 remained clustered in isolated patches on the plasma membrane, regardless of whether the nerve terminals were subjected to strong stimulation (Willig et al., 2006). This finding indicates that the Syt1 clusters on an SV do not lose their identity or laterally disperse at the plasma membrane during SV recycling. Although it remains unclear whether this behavior is representative of that of all proteins localized to the SV, it is possible that many other SV proteins follow the same path as Syt1 to some extent, as SV proteins were reported to form detergent-resistant complexes (Bennett et al., 1992). Alternatively, as shown for the plasma membrane SNARE protein Syntaxin 1A, it is also plausible that specific lipid-protein interactions, cholesterol-based lipid rafts, or homomeric SNARE motif interactions play a role in clustering SV cargos (Lang et al., 2001; Gil et al., 2005; Sieber et al., 2006; van den Bogaart et al., 2011). The contributions of other possible factors to preserve protein clusters on SVs, e.g., a luminal matrix that “bridges” SV residents, have not yet been clarified.

In contrast, a large body of research using exogenous pHluorin-based SV cargo probes and fluorophore-coupled antibodies, which enable the labeling of endogenous SV proteins, suggests that SV proteins inserted into the plasma membrane upon exocytosis rapidly diffuse away from the release sites and mix with SV proteins present on the plasma membrane before endocytosis (Sankaranarayanan and Ryan, 2000; Li and Murthy, 2001; Granseth et al., 2006; Wienisch and Klingauf, 2006). Furthermore, instead of newly exocytosed SV proteins, a fraction of SV proteins that pre-exist on the plasma membrane is preferentially retrieved by compensatory endocytosis (Wienisch and Klingauf, 2006). Notably, fluorescence nanoscopy of surface-labeled Syt1 showed perisynaptic localization of Syt1 clusters, presumably representing a “hot spot” for endocytosis (Hua Y. et al., 2011). In addition, high-resolution time-lapse imaging of newly exocytosed SV proteins revealed that upon exocytosis, SV proteins rapidly disperse (Gimber et al., 2015). However, these proteins are largely confined within the presynaptic bouton, and subsequently slowly recluster at the periactive zone. Altogether, it is conceivable that a pre-sorted and pre-clustered pool of SV proteins (named the readily retrievable pool of SV proteins [RRetP]; Hua Y. et al., 2011) on the presynaptic membrane might be responsible for the initial retrieval of SV proteins into endocytosed vesicles. During repetitive stimulations through which the RRetP are exhausted, the SV proteins inserted into the plasma membrane by exocytosis travel to the “hot spot” by diffusion and are likely retrieved by subsequent endocytosis.

## Retrieval signals within SV proteins for clathrin-mediated SV reformation

Regardless of endocytosis modes, new SVs appear to be reformed either from the plasma membrane or eventually from endosomal compartments presumably via the clathrin-mediated pathway, and various adaptor proteins that selectively recognize individual cargo are required to recruit SV cargo into newly generated SVs (Saheki and De Camilli, 2012). These adaptors can bind directly to cargo proteins, while simultaneously recruiting the clathrin coat proteins. As outlined in Introduction, among the adaptor proteins, AP-2 might be play a central role in the recognition of SV cargos at the plasma membrane, enabling the selective retrieval of SV cargo proteins and preventing unexpected contamination of plasma membrane residents (Traub and Bonifacino, 2013; Paczkowski et al., 2015). Other adaptor protein complexes, such as AP-1 and AP-3, may play similar roles in SV cargo recognition, presumably at different sites, such as endosomes (Kokotos and Cousin, 2015). Furthermore, for some SV cargos, other accessory proteins, such as the AP-180/CALM family, endophilin, and stonin, likely contribute to cargo selection and efficient retrieval (Voglmaier et al., 2006; Kononenko et al., 2013; Kaempf et al., 2015; Koo et al., 2015). Finally, specific or non-selective cargo–cargo interactions within SVs may contribute to cargo selection into newborn SVs (Pan et al., 2015; Gordon et al., 2016; Rajappa et al., 2016).

From a quantitative perspective, the average synapse contains 1,000–2,000 AP-2 subunits, ~3,000 AP-180, and ~2,000 endophilins, whereas it contains 5–10 times more SV cargos (Wilhelm et al., 2014). Therefore, the total molecular components responsible for CME would capture only 10–20% of all the cargo simultaneously, implying that the number of adaptor proteins would limit the efficient retrieval of SV cargos by CME upon high-frequency repetitive stimulation. This may explain why CME is saturable and SV reformation via ADBE is adopted to deal with excess SV cargo during periods of repetitive stimulation (Sankaranarayanan and Ryan, 2000; Clayton et al., 2008; Kononenko et al., 2014).

In the following sections, we will present amino acid sequences or motifs within SV proteins that are responsible for SV cargo recognition by the adaptor proteins that underlie the efficient retrieval of individual cargo into clathrin-coated vesicles. We will further describe the contribution of other endocytic motifs, as well as specific cargo–cargo interactions that affect the efficiency of SV protein retrieval.

### Di-leucine motif

**Figure 1:**
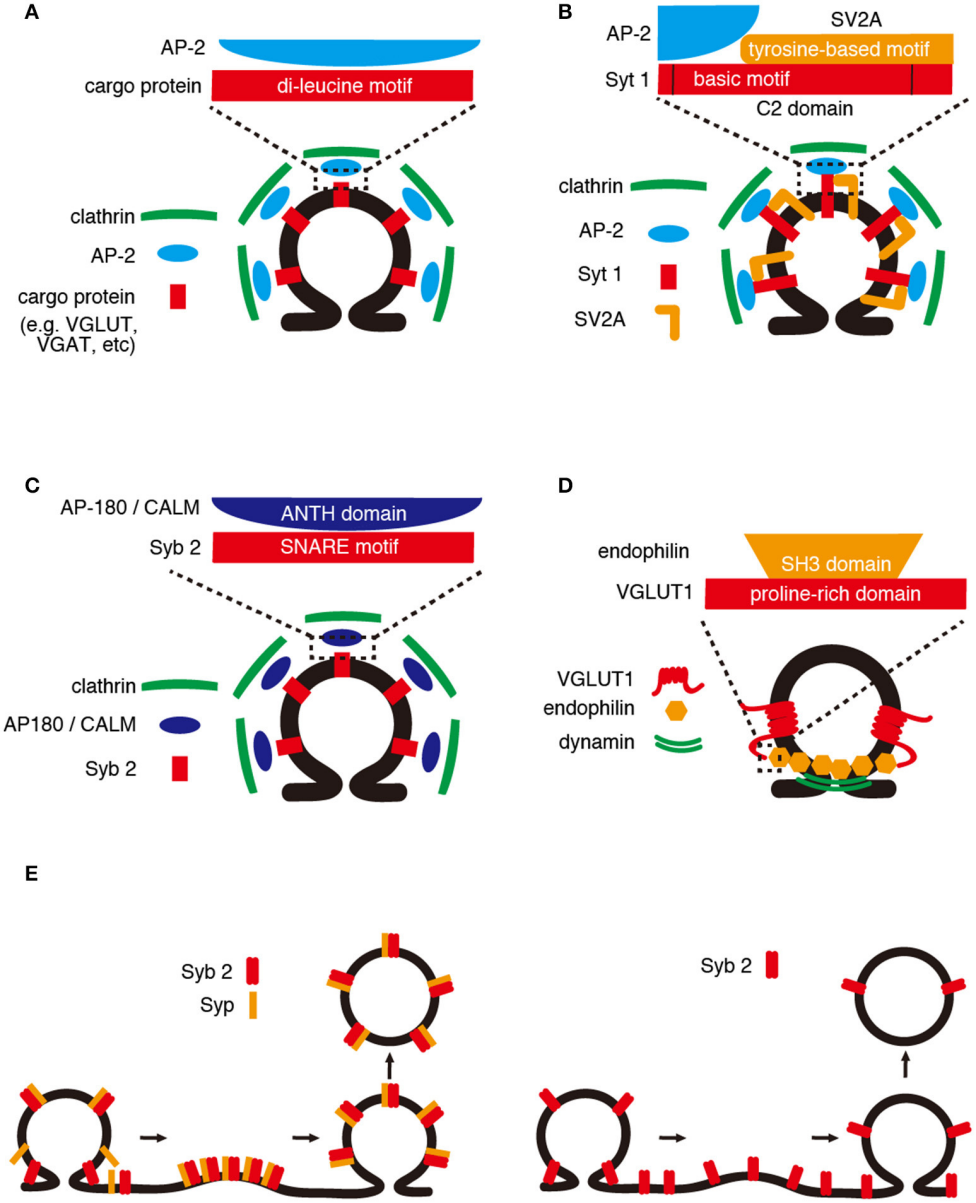
Retrieval mechanisms of SV cargos. **(A)** Di-leucine motif. AP-2 recognizes a di-leucine or a dileucine-like motif within the cytoplasmic region of SV cargos (e.g., VGLUT and VGAT). AP-2/cargo protein complexes are incorporated into SV surrounded by clathrin complexes. **(B)** C2 domain. The basic motif within the C2 domains of Syt 1 is required for binding of AP-2, and the binding is enhanced by a tyrosine-based motif of SV2A. **(C)** SNARE motif. The SNARE motif of Syb2 binds to the ANTH domain of AP180/CALM, which recruits clathrin complexes. **(D)** Proline-rich domain. The proline-rich domain of VGLUT1 interacts with a SH3 domain of endophilin, which senses membrane curvature and recruits dynamin that mediates membrane tubulation and fission. **(E)** Cargo–cargo interaction. (Left panel) Syp and Syb2 form a 1:2 complex on SVs, and the complex is dissociated upon SNARE complex assembly during exocytosis. After exocytosis, the Syp/Syb2 complex is reformed and incorporated into SV. (Right panel) In the absence of Syp, the efficiency of Syb2 retrieval would be decreased.

A di-leucine motif, known as one of the well-conserved endocytic motifs, is represented by [E/D]xxxL[L/I] (Kelly et al., 2008). The motif can be recognized by the AP-2 adaptor complex, which enables the connection of cargo proteins to clathrin coats (Figure 1A). Many abundant SV proteins, such as Syt1, Syb2, and all vesicular neurotransmitter transporters, contain typical or atypical di-leucine-like motifs in their cytoplasmic tails. These motifs are required, and are sufficient in most cases, to target these cargos into small synaptic vesicle-like microvesicles (SLMVs) in neuroendocrine cells (e.g., PC12 cells) (Grote and Kelly, 1996; Tan et al., 1998; Blagoveshchenskaya et al., 1999). Furthermore, phosphorylation and acidic residues located upstream of the di-leucine motifs in the vesicular acetylcholine transporter and vesicular monoamine transporter 2 dictate sorting of the transporters to distinct secretory organelles in PC12 cells, highlighting the importance of negative charges within the motifs (Krantz et al., 2000). In addition, di-leucine motifs help prevent the accumulation of stranded cargos in the plasma membrane, collectively suggesting the important roles of these motifs in directing the cargo to SLMVs, as well as in the recruitment of cargo from the plasma membrane (Tan et al., 1998).

In neurons, the role of di-leucine motifs in the retrieval of vesicular amino acid transporters has been intensively investigated using pHluorin-based probes. First, VGLUT1 contains an atypical di-leucine motif at the C-terminal cytoplasmic tail, and similar sequences are present in VGLUT2 and VGLUT3 (Voglmaier et al., 2006). Replacing the hydrophobic residues in the motif with alanine or glycine results in a significant reduction in the rate of VGLUT1 internalization following repetitive stimulation (e.g., 5 Hz, 60 s) (Voglmaier et al., 2006; Foss et al., 2013; Pan et al., 2015). Further, VGLUT1 contains two di-leucine-like motifs at the N terminus, which are not well-conserved in the other isoforms. In the absence of all three motifs, VGLUT1 becomes stranded at the cell surface, no longer responds to stimulation, and is not targeted to SVs. Notably, the C-terminal motif facilitates the retrieval of VGLUT1 via AP-2, whereas the N-terminal motifs use AP-1 dependent pathways, indicating that VGLUT1 retrieval is mediated by two distinct mechanisms involving different adaptor proteins (Foss et al., 2013). In contrast to VGLUT1, mutations in the C-terminal di-leucine-like motif in VGLUT2 led to increased surface expression (>10-fold more than wild-type VGLUT2) and disrupted synaptic targeting of VGLUT2 (Foss et al., 2013). Thus, although VGLUT2 recycling is almost exclusively dependent on the C-terminal di-leucine-like motif, VGLUT1 also relies on its N-terminal trafficking motifs. Second, putative trafficking motifs of vesicular GABA transporter (VGAT: also referred to as VIAAT) have been examined (Santos et al., 2013). Like VGLUTs, VGAT contains an atypical di-leucine motif (E_39_EAVGFA_45_), consisting of acidic residues at the −4 and −5 positions upstream of two hydrophobic residues, and the F_44_A/AA mutation resulted in reduced retrieval of VGAT during and after repetitive stimulation. Interestingly, the E_39_E_40_/GG mutation also caused substantial delay of VGAT retrieval, underscoring the importance of acidic residues in the di-leucine-like motif. Moreover, single mutations in the acidic residues led to a shift from the AP-2-dependent pathway to the AP-3-dependent pathway. These results revealed the following: (1) hydrophobic residues are more compatible and di-leucine motifs are more diverse than previously envisioned and (2) acidic residues in the di-leucine motif may play an essential role in selective binding to specialized adaptor proteins, including AP-1/2/3 (see also Proline-rich domain, below).

Altogether, although the detailed roles of the di-leucine-like motifs of Syt1, Syb2, and other vesicular neurotransmitter transporters (VMATs/VAChT) in recycling at the presynaptic terminal remain to be explored, the existence of di-leucine-like motifs in the major SV cargos indicate a vital role in their retrieval. This sequence also predicts which adaptor protein complexes are involved during recognition, and can ultimately define the distribution of the SV cargos across SV pools with distinct release properties.

### C2 domains

Some members of the Syt family are known to be Ca^2+^ sensors for rapid, synchronized SV exocytosis. Syts are single-transmembrane proteins with cytoplasmic domains that harbor tandem C2 domains (C2A and C2B) that interact with Ca^2+^, SNAREs, and phospholipids. While the C2B domain appears to be essential for rapid Ca^2+^-dependent exocytosis (Mackler et al., 2002; Nishiki and Augustine, 2004), it also functions as a high affinity receptor for the μ-subunit of the AP-2 complex (Zhang et al., 1994), indicating its role in endocytosis. Within the C2B domain, two adjacent lysine residues, known as the “basic motif,” are required and sufficient for Ca^2+^-dependent oligomerization of Syt1 (Chapman et al., 1998) and AP-2 binding (Haucke et al., 2000). The basic motif comprising the putative AP-2 binding site is highly conserved among other Syt family members in mammals (Grass et al., 2004). Intriguingly, the interaction can be inhibited by a peptide containing the basic motif, but not by one containing a classical di-leucine motif (Grass et al., 2004), suggesting that the basic motif is responsible for recognition of Syt1 by AP-2 (Figure 1C). In addition, binding between the basic motif of Syt1 and AP-2 can be markedly enhanced in the presence of a tyrosine-based endocytic motif (YxxΦ, where Y, x, and Φ denote a tyrosine, polar residue, and large hydrophobic side chain, respectively; Haucke and De Camilli, 1999). This endocytic motif is presumably conferred by another SV protein, SV2A (see also below, *Cargo–cargo interactions*). A tyrosine-based motif containing peptides can simultaneously induce AP-2 and clathrin coat assembly, facilitating clathrin/AP-2 recruitment onto protein-free liposomes *in vitro* (Haucke and Krauss, 2002). These findings collectively indicate that the basic motif of Syt1 is not only responsible for Syt1 retrieval during endocytosis but also plays a key role in concentrating the endocytic protein machinery at the “hot spot” of endocytosis, upon the elevation of cytoplasmic [Ca^2+^] near release sites.

In addition to the Ca^2+^-dependent direct binding of Syt1 to AP-2, another accessory protein named stonin 2 can connect Syt1 to AP-2, in a Ca^2+^-independent manner. Stonin 2, whose drosophila ortholog was originally identified as one of the temperature-sensitive immobile Drosophila mutants, has a motif with the consensus sequence WVxF at the N terminus (Maritzen et al., 2010). This sequence is responsible for binding to the AP-2 α-adaptin ear domain and μ2 subunit (Walther et al., 2004). The protein can also bind to the C2 domain of Syt1 through the C-terminus μ-homology domain (μ-HD), establishing another physical link between Syt1 and AP-2 (Martina et al., 2001; Walther et al., 2004). The μ-HD of stonin 2 was necessary for the sorting of stonin 2 to the presynaptic terminals in cultured hippocampal neurons and to the plasma membrane upon co-expression with Syt1 in a neuroblastoma cell line (Walther et al., 2004). In addition to the biochemical and genetic evidence supporting a possible role of stonin 2 in the selective retrieval of Syt1 during endocytosis, the retrieval kinetics of both Syt1 and Syp were faster in stonin 2-deficient neurons, which was only observed when high-frequency stimulation (40 Hz) was applied (Kononenko et al., 2013). In contrast, the deletion of stonin 2 resulted in the selective accumulation of Syt1 on the cell surface, indicating that it was required for maintaining sufficient copies of Syt1 during constitutive synaptic vesicle recycling, and not for the rapid retrieval of Syt1 upon stimulation.

Elucidating the contributions of the C2 domains of Syt1 to its retrieval has proven difficult, mainly because Syt1 plays an essential role not only in Ca^2+^-triggered exocytosis, but also in SV endocytosis (Jorgensen et al., 1995; Poskanzer et al., 2003, 2006; Nicholson-Tomishima and Ryan, 2004; Yao et al., 2011). Therefore, the interaction might be central to the initiation of SV endocytosis through recruitment of clathrin and accessory proteins that are essential for SV reformation.

### SNARE motif

Syb2, the most abundant SV protein and major SNARE on SVs, is essential for Ca^2+^-triggered rapid SV fusion to the plasma membrane. Although Syb2 is abundant (~70 copies per SV) and also present on the presynaptic plasma membrane as a potential RRetP, reduced expression of Syb2 might have a substantial impact on synaptic transmission (Koo et al., 2015).

Syb2 is a small single-span transmembrane protein, in which the “SNARE motif” predominates the cytoplasmic domain (~70 of 90 amino acids). The SNARE motif of Syb2 is critical for the formation of the SNARE complex with syntaxin 1 and SNAP-25 at the plasma membrane (Jahn and Scheller, 2006). However, several studies have indicated that the motif also works as a retrieval signal during endocytosis. Genetic data in *Caenorhabditis elegans* (Nonet et al., 1998; Dittman and Kaplan, 2006) and *Drosophila* (Zhang et al., 1998; Bao et al., 2005) demonstrated that the AP-180 N-terminal homology (ANTH) domain-containing endocytic protein AP180 is associated with proper SV sorting of Syb2. In mammals, the ANTH domain family consists of two family members, CALM and AP180. Biochemically, AP180 and CALM can bind to PtdIns(4,5)P_2_ and clathrin simultaneously, enabling membrane tethering of clathrin. Moreover, AP180 (and presumably CALM) can not only induce clathrin lattice formation in lipid monolayers *in vitro*, but also facilitate the formation of clathrin-coated pits in the presence of AP-2 (Ford et al., 2001).

In addition to their pivotal function in recruiting the endocytic machinery, both AP180 and CALM bind to the N-terminal half of the Syb2 SNARE motif through their ANTH domain (Figure 1D). Interestingly, knockdown of either CALM or AP180 induced higher surface expression of Syb2-pHluorin, but not VGLUT1-pHluorin. The simultaneous knockdown of both further increased the surface expression, suggesting their co-operative roles in the selective retrieval of Syb2 (Koo et al., 2011). Further, mutations in Syb2-pHluorin, which diminished its binding to the ANTH domain, resulted in a marked increase in its surface expression (~70%), thus supporting the important roles of CALM and AP180 in the selective retrieval of Syb2. Recent research regarding knockout (KO) of AP180 in mice substantiated its role in Syb2 retrieval because surface expression of Syb2-pHluorin, but not Syt1-pHluroin, was increased. Moreover, the endocytosis of Syb2-pHluorin, but not Syt1-pHluorin, was diminished (Koo et al., 2015). Collectively, these findings suggest that, in accordance with a cooperative contribution of Syp binding to Syb2 (Adams et al., 2015) (see also below, *Cargo–cargo interactions*), binding of the N-terminal SNARE motif of Syb2 to the AP180 family might play a key role in the selective retrieval of Syb2.

### Proline-rich domain

A proline-rich domain (PRD) is a protein domain enriched in proline residues. PRDs are known to interact with various proteins through their src-homology 3 domains (SH3 domains), and these interactions mediate the assembly of specific protein complexes. The endocytic protein dynamin has a crucial role in membrane fission during vesicle formation. It contains a C-terminal PRD which interact with the SH3 domains of various endocytosis proteins, such as endophilin (Ringstad et al., 1999; Gad et al., 2000). VGLUT1 also contains PRDs at its C-terminus that interact with the SH3 domain of endophilin A family proteins (Figure 1B; De Gois et al., 2006; Vinatier et al., 2006; Voglmaier et al., 2006). Endophilins contain an N-terminal Bin-Amphiphysin-Rvs (BAR) domain, which is implicated in the generation of membrane curvature (Farsad et al., 2001). Considering its association with dynamin, also via its SH3 domain, endophilin is thought to recruit dynamin to endocytic sites where the BAR domain may sense the membrane curvature, during the initial stage of vesicle endocytosis (Ringstad et al., 1999; but see Milosevic et al., 2011, suggesting a role of endophilin in the uncoating of clathrin after fission of endocytosed SVs). Whether endophilin is involved in clathrin-dependent endocytosis (CME) or in clathrin-independent endocytosis (CIE) remains under intense debate (Ringstad et al., 1999; Milosevic et al., 2011). Nevertheless, VGLUT1-pHluorin lacking PRD (VGLUT1-ΔPRD), which is thereby incapable of binding to endophilin, exhibited slower retrieval from the plasma membrane only at the late phase of prolonged repetitive stimulation (e.g., 5 Hz, 300 s) (Voglmaier et al., 2006; Pan et al., 2015). However, the delay was less pronounced compared with that observed with mutations in the di-leucine-like motif of the VGLUT1 C-terminus, which was apparent after a shorter stimulation protocol (5 Hz, 60 s). Notably, inhibition of the AP-3 pathway by brefeldin A restored the rate of VGLUT1-ΔPRD retrieval, suggesting that the retrieval of VGLUT1 depends both on AP-2 and AP-3 and that endophilin may recruit VGLUT1 to the faster AP-2 pathway (Voglmaier et al., 2006). It should be noted that VGLUT2-pHluorin, which intrinsically lacked a PRD at its C-terminus, exhibited slower decay kinetics than VGLUT1-pHluorin (Foss et al., 2013). It remains to be clarified whether the existence of a PRD in VGLUT1 alone resulted in the differences between the isoforms.

In addition to the role of the PRD in VGLUT1 retrieval, this domain may be involved in the regulation of release probability (Weston et al., 2011). Overexpression of endophilin in hippocampal neurons increased the release probability, whereas suppression of endophilin had the opposite effect. Notably, the increase in release probability induced by exogenous endophilin was mediated by the BAR domain of endophilin. Moreover, the effect could be “buffered” by the PRD of VGLUT1, indicating that the PRD in VGLUT1 may regulate available endophilin molecules on SVs that increase the release probability.

Syb2 also contains a PRD at its N-terminus. Although its role in Syb2 retrieval has not been directly investigated, both disruption of the Syb2 gene and acute injection of Syb2 N-terminal peptide (1–26 containing PRD) into the presynaptic terminal of the calyx of Held significantly reduced endocytosis itself (Deák et al., 2004; Hosoi et al., 2009). Moreover, the PRD of Syb2 is involved in efficient SV recruitment to release sites (Wadel et al., 2007). Thus, the PRD of Syb2 plays a pivotal role in SV recycling rather than Syb2 retrieval exclusively.

### Cargo–cargo interactions

Apart from the endocytic motifs of major SV cargos that play a crucial role in their retrieval, described above, other interactions (both specific and non-specific) among SV cargos might play a role in their cooperative retrieval. In fact, major SV proteins were shown to form distinct protein complexes, depending on the detergents used for solubilization (Bennett et al., 1992), a finding suggesting their multiple interactions *in vivo*. Moreover, the construction of a 3D model of an average SV revealed that the SV is a protein-rich structure with one quarter of the membrane area occupied by the transmembrane domains of SV cargo proteins (Takamori et al., 2006). This finding indicated that non-selective hydrophobic interactions, as well as specific sequence-dependent interactions between cytoplasmic tails of individual cargos, are likely to occur in an SV. There is now evidence to support the notion that direct interactions among SV cargos might also significantly influence their cooperative retrieval, as described below.

SV2, which is structurally similar to sugar transporters and functionally implicated in epilepsy (Crowder et al., 1999; Janz et al., 1999; Lynch et al., 2004), binds specifically to the C2B domain of Syt1 through its N-terminus cytoplasmic tail. This binding, which may or may not be Ca^2+^-dependent (Schivell et al., 1996; Lazzell et al., 2004), depends on the phosphorylation of the Thr84 residue of SV2A by the casein kinase 1 family (Pyle et al., 2000; Zhang et al., 2015). Although the mutagenesis of T84 in SV2A showed a subtle effect on SV2A recycling, abolishing the phosphorylation-dependent SV2A interaction with Syt1 by mutagenesis of the C2B domain of Syt1(K326/328A) and shRNA knockdown of SV2A, resulted in a faster retrieval of Syt1-pHluorin, but not Syp-pHluorin (Kaempf et al., 2015). Further, disruption of the tyrosine-based motif in the SV2A N-terminus (Y^46^SRF) resulted in a higher proportion of Syt1 on the plasma membrane (Yao et al., 2010). These results collectively indicated that SV2A might function as a molecular chaperone of Syt1, limiting the retrieval of Syt1 during endocytosis.

Syp and Syb2, the two most abundant proteins on SVs, are known to form a heterodimer complex, probably via the labile interactions between the transmembrane domains of the two proteins (Edelmann et al., 1995; Washbourne et al., 1995). In the presence of cholesterol, the complex is stable and can be purified from native brains (Adams et al., 2015). Consistent with biochemical data, the structure of the complex analyzed by single particle three-dimensional EM reconstruction revealed that Syp and Syb2 assembled into a hexameric ring structure, wherein 6 Syp molecules formed the basis of the ring structure, with 6 Syb2-dimers located in between (Arthur and Stowell, 2007; Adams et al., 2015). This stoichiometry resembles that of those proteins in purified SVs (~30 Syp and ~70 Syb2/SV) (Takamori et al., 2006); if such were the case, a single SV would contain 5–6 Syp/Syb2 complexes. However, the extent of complex formation is modulated under various conditions (Becher et al., 1999; Khvotchev and Südhof, 2004), suggesting that the formation of the complex must be controlled by dynamic regulation during neuronal activities. Despite the critical role of Syb2 in SV exocytosis, i.e., mediating the final step of membrane fusion of SVs to the plasma membrane, the presynaptic function of Syp is largely unknown, although subtle alterations in short-term plasticity in Syp-deficient neurons was previously reported (McMahon et al., 1996). Recently, however, it was demonstrated that Syb2 retrieval was markedly reduced in Syp-KO neurons, especially after strong repetitive stimulation (200 APs at 10 Hz). The retrieval of other SV cargo proteins, such as VGLUT1 and Syt1, however, was only mildly affected during the late phase of retrieval (Figure 1E; Gordon et al., 2011). As the global turnover of SVs in Syp-KO neurons was intact, it appears that Syp plays a specific role in the retrieval of Syb2 during endocytosis. An independent study using Syp-KO mice, however, revealed not only that retrieval of Syt1-pHluorin and SV2A-pHluorin was delayed, but also that compensatory endocytosis after repetitive stimulation was impeded in Syp-KO neurons. These results indicated a role for Syp in SV membrane endocytosis, rather than the selective retrieval of Syb2 into newborn SVs (Kwon and Chapman, 2011). More recently, another study using Syp-KO neurons demonstrated that Syp supported the NSF-dependent disassembly of Syb2 clustering at the release sites. The loss of Syp resulted in remarkable short-term depression (measured by a Syb2-pHluorin probe), suggesting that Syp-Syb2 interaction is essential for efficient Syb2 clearance from the release sites upstream of endocytosis, but not for its retrieval (Rajappa et al., 2016). Consistent with these findings, imbalance in the levels of Syp and Syb2 resulted in alterations in the surface expression of Syb2 (Gordon et al., 2016; Rajappa et al., 2016), strongly indicating that Syp levels might affect presynaptic Syb2 localization and targeting to SVs. Therefore, in addition to Syb2 interaction with AP180/CALM via the SNARE motif (Figure 1D), its interaction with Syp contributes to the efficient retrieval of Syb2.

In addition to the critical roles of specific cargo–cargo interactions in their retrieval described above, relatively non-selective cargo–cargo interactions may occur during retrieval. For instance, removal of VGLUT1, as well as impairment of VGLUT1 retrieval through mutation of the di-leucine motif, impeded the retrieval of other cargos, such as SV2A-pHluorin, Syp-pHluorin, and Syb2-pHluorin, but not Syt1-pHluorin (Pan et al., 2015). As VGLUT1-pHluorin exhibits the fastest decay kinetics after stimulation among the pHluorin-based probes, these observations indicated that VGLUT1 somehow orchestrates the recruitment of other SV proteins into newborn SVs. Interestingly, VGLUT1-pHluorin lacking both the di-leucine-like motif and PRD at its C-terminus had no effect on the retrieval kinetics of SV2A-pHluorin and Syp-pHluorin, indicating that VGLUT1 dictates the retrieval of other SV cargos via its PRD (Pan et al., 2015). However, in the presence of “slow” VGLUT1-pHluorin lacking the di-leucine-like motif, both SV2-pHluorin and Syp-pHluorin exhibited faster retrieval kinetics than the mutant VGLUT1-pHluorin, indicating that other regulatory mechanisms to control retrieval must be present independent of VGLUT1 (Pan et al., 2015).

These observations suggest that VGLUT1 dictates the retrieval of other SV cargos, but how does the expression of VGLUT1 affect the retrieval of other SV cargos? First, chronic alterations in VGLUT1 levels may alter the protein composition of SVs and endosomes, including trafficking proteins, such as rab3, rab5, and synapsin (Fremeau et al., 2004; Siksou et al., 2013). Moreover, gene disruption of VGLUT1 in mice resulted in the flattening of SVs under certain fixation conditions, as well as the accumulation of endosomal tubular structures, with concomitant changes in the expression levels of endosomal proteins, indicating that expression of VGLUT1 may support a recycling pathway. Furthermore, because VGLUTs concentrate glutamate in SVs, and also exhibit Cl^−^ conductance, the function of VGLUT as a transporter may result in osmotic imbalances across the SV membrane (Maycox et al., 1988; Tabb et al., 1992; Schenck et al., 2009; Preobraschenski et al., 2014; Eriksen et al., 2016). It remains largely unknown whether these physical changes in SVs resulting from the functions of VGLUTs have any influence on the clustering of other SV proteins. Finally, it remains to be seen whether similar mechanisms by which transporters play central roles in coordinating retrieval of other SV cargos exist in different vesicle populations carrying different vesicular neurotransmitter transporters, including other VGLUT isoforms.

## Cargo retrieval during clathrin-independent endocytosis

As described earlier, multiple modes of membrane retrieval exist. Morphological evidence has revealed that unlike clathrin-coated vesicles that are predominantly generated at the plasma membrane after mild stimulation (e.g., 5 Hz), clathrin-free large ELVs accumulate after high-frequency repetitive stimulation (e.g., 40 Hz) during ADBE (Heuser and Reese, 1973; Clayton et al., 2008; Kononenko et al., 2014). This finding suggests that SV cargos should be sorted into these endosomes. Although clathrin-coated vesicles are subsequently formed from the ELVs (Takei et al., 1995; Kononenko et al., 2014), whether all SV cargos are incorporated into the ELVs remains unclear. The major discrepancy regarding SV cargo retrieval upon high frequency repetitive stimulations arose from experiments in which CME was inhibited. First, there are clear indications that disruption of CME during 40 Hz stimulation, e.g., knock-down of AP-2 subunits or clathrin heavy chain, resulted in a slowdown of decay kinetics of pHluorin-based probes, arguing that CME is a predominant pathway for most SV cargos (Granseth et al., 2006; Nicholson-Fish et al., 2015). With the same stimulation, disruption of ADBE by syndapin 1 knockdown does not affect the retrieval of Syp (Clayton et al., 2009; Nicholson-Fish et al., 2015), indicating that Syp is not retrieved through ADBE. Notably, VAMP4-pHluorin, a homolog of VAMP2/Syb2, is selectively retrieved into vesicles generated by ADBE, which are slowly re-acidified presumably owing to their large volume-to-surface ratio (Nicholson-Fish et al., 2015; Okamoto et al., 2016). Further, the retrieval of VAMP4 during ADBE depends on a di-leucine motif of VAMP4, and knockdown of VAMP4 abolished dextran uptake by ADBE, indicating that VAMP4 is not only an ADBE-specific cargo but is also essential for ADBE. In contrast, recent studies have reported that the endocytic retrieval of SV cargos upon similar stimulation was largely unaffected by treatments that blocked CME (Kim and Ryan, 2009; Kononenko et al., 2014; Wu et al., 2014). In this scenario, SV cargos are retrieved into ELVs and subsequently sorted into SVs in a clathrin-dependent manner.

Another type of CIE is UFE, which involves rapid invagination and pinch-off of larger vacuole-like membrane structures within 50 ms. UFE was identified when cultured hippocampal neurons were stimulated optogenetically and rapidly frozen for electron microscopic studies (Watanabe et al., 2013). This mode of endocytosis was predominant when experiments were performed at physiological temperatures (~35°C) (Watanabe et al., 2014). These morphological observations were subsequently supported by high-resolution membrane capacitance measurements in hippocampal and cerebellar mossy fiber terminals in which single action potentials triggered very rapid (τ ~470 ms) clathrin-independent/dynamin-dependent membrane retrieval (Delvendahl et al., 2016).

When SV cargo retrieval was assessed at physiological temperature, the retrieval of pHluorin-based probes (Syt1 and Syp, among others) in cultured hippocampal neurons was largely clathrin-independent, but dependent on formin-dependent actin assembly (Soykan et al., 2017). Considering that clathrin appears to be dispensable for membrane retrieval at physiological temperatures and that re-acidification kinetics may be rate-limiting for the decay of pHluorin fluorescence (Egashira et al., 2015; Nicholson-Fish et al., 2015; Okamoto et al., 2016), the previously reported involvement of clathrin-dependent retrieval motifs at room temperature with the use of pHluorin probes should be carefully reconciled in future experiments.

## Concluding remarks

In this report, we have summarized our current knowledge regarding the molecular signatures of SV proteins, which affect the efficiency of their retrieval. Some motifs are cargo-specific, but some interact with multiple adaptor proteins, presumably with different kinetics. This complexity of cargo-adaptor interactions may be responsible for the fidelity and efficiency of SV cargo retrieval within a wide range of synaptic activity. In addition, some SV cargos, such as VGLUTs, Syb2, and Syt1, contain multiple retrieval motifs that are recognized by different adaptor proteins. As SV membranes are reformed via multiple modes at different speeds depending on neural activity, the existence of multiple motifs in individual cargos may represent a “safeguard” system to maintain the amount of essential SV cargo at any time. Future studies will be needed to proceed toward a complete mechanistic understanding of SV cargo retrieval.

## Author contributions

YM prepared initial drafts of the manuscript and the figure and ST finalized them.

### Conflict of interest statement

The authors declare that the research was conducted in the absence of any commercial or financial relationships that could be construed as a potential conflict of interest.
